# Evaluating the risk factors for the development of benign disorders of defaecation: a surgical perspective

**DOI:** 10.1007/s10151-023-02843-w

**Published:** 2023-07-27

**Authors:** P. Chaichanavichkij, M. Hartmann, S. M. Scott, N. Fenton, C. H. Knowles, Disorders of Defaecation Delphi Group, E. V. Carrington, C. Chew, A. Curry, K. Gorissen, S. Morris, S. Siddiqi, A. Williams

**Affiliations:** 1 National Bowel Research Centre and GI Physiology Unit, Centre for Neuroscience, Surgery & Trauma, Blizard Institute, Queen Mary University of London, 1st Floor, Abernethy Building, 2 Newark Street, London, E1 2AT UK; 2 Risk and Information Management Research Group, School of Electronic Engineering and Computer Science, Queen Mary University of London, London, UK

**Keywords:** Risk factors, Faecal incontinence, Constipation, Delphi

## Abstract

**Purpose:**

There remains uncertainty as to which risk factors are important for the development of defaecatory problems as a result of heterogeneity of published evidence. Understanding the impact of risk factors may be important in selecting targets for disease prevention or reversal. The aim of this study was to identify and evaluate risk factors for faecal incontinence and chronic constipation.

**Methods:**

Risk factors for chronic constipation and faecal incontinence were long-listed from scientific literature, then anonymously evaluated (by 50 predominantly colorectal surgical experts from the UK Pelvic Floor Society) using a Delphi technique. Each risk factor was rated as independent, a co-factor, or not a risk factor. Independent risk factors were rated between 1 (not important) and 10 (critically important) with mean (± standard deviation) calculated.

**Results:**

Thirty-eight risk factors for chronic constipation were evaluated. Eighteen were classed as independent and 16 as co-factors. Opioid analgesia (7.87 ± 2.05), eating disorders (7.80 ± 1.72), and history of abuse (7.70 ± 1.89) were scored as most important independent risk factors. Female sex (6.60 ± 2.02) was considered an independent risk factor but increasing age was rated a co-factor. Thirty-three risk factors for faecal incontinence were evaluated. Twenty were classed as independent and eight as co-factors. Third- or fourth-degree tear (8.88 ± 1.57), instrumental delivery (8.47 ± 1.58), and grand multiparity (8.00 ± 1.63) were rated most important. Increasing age (7.41 ± 2.14) and female sex (7.58 ± 2.05) were both considered independent risk factors.

**Conclusions:**

Several risk factors for chronic constipation and faecal incontinence were selected by Delphi approach. These factors will feed forward into Bayesian models of disease prediction that combine data and expert knowledge.

**Supplementary Information:**

The online version contains supplementary material available at 10.1007/s10151-023-02843-w.

## Introduction

Faecal incontinence and chronic constipation are common disorders of defaecation, with faecal incontinence affecting around 6% [1], and chronic constipation affecting between 10% and 15% of the population, depending on diagnostic criteria used [2]. Both conditions have impact on quality of life [3], resulting in significant health burden [3, 4]. While advances have been made in the assessment of anorectal function, which has improved the understanding of the pathophysiology underlying disorders of defaecation [5], better understanding of risk factors may reveal further insights into pathophysiology, direct future research, and, most importantly, highlight targets for disease prevention or modification.

Several risk factors for developing faecal incontinence and chronic constipation have been identified, predominantly through cross-sectional studies which vary in the definitions used, methodology, study population, and potential risk factors assessed. These limitations prevent aggregation of results by meta-analysis [6].

The Delphi technique is an effective method for arriving at consensus on broad and complex problems [7, 8]. Using knowledge and experience of experts (primarily colorectal surgeons) in the field of pelvic floor disorders, in combination with contemporary published evidence, this Delphi study aimed to identify and evaluate risk factors for faecal incontinence and chronic constipation as a preliminary step toward building Bayesian models of disease prediction that combine data and expert knowledge.

## Methods

### Delphi methodology

The Delphi technique is characterised by four methodological features: expert participants, iterative rounds of enquiry, a dependency of the design of subsequent rounds on the basis of the response of the previous round, and anonymous participation [9, 10].

#### Expert participants

Participants were recruited voluntarily from members of The Pelvic Floor Society (TPFS), a UK subspecialty society affiliated to the Association of Coloproctology of Great Britain & Ireland. Email invitations to take part in the survey in May 2021 were sent to the whole membership (399 members) in April 2021. TPFS membership criteria ensured the selection of current specialists in pelvic floor disorders.

#### Rounds of enquiry and dependency of the design of subsequent rounds

**Fig. 1:**
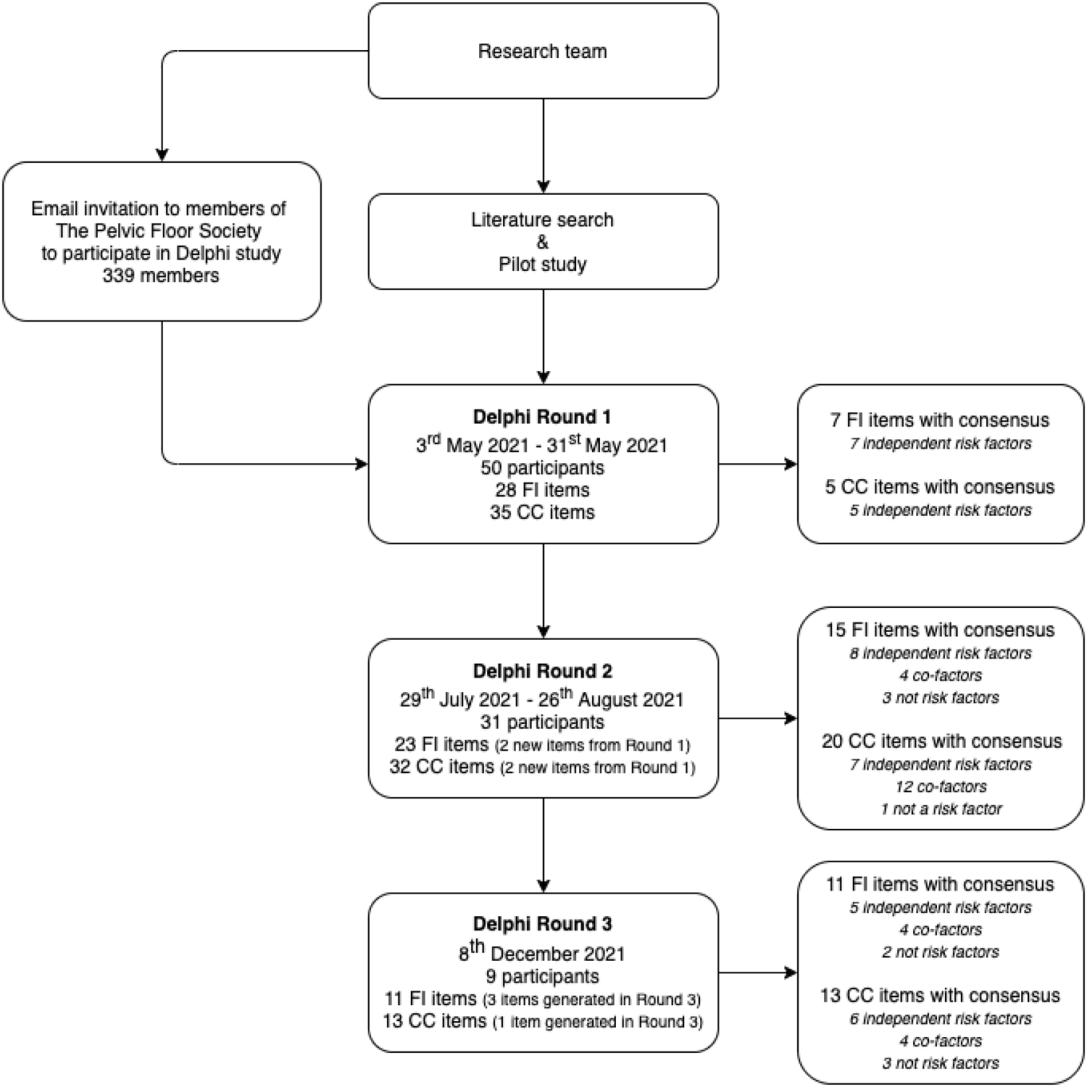
Delphi study schema. *FI*, faecal incontinence; *CC*, chronic constipation

**Fig. 2:**
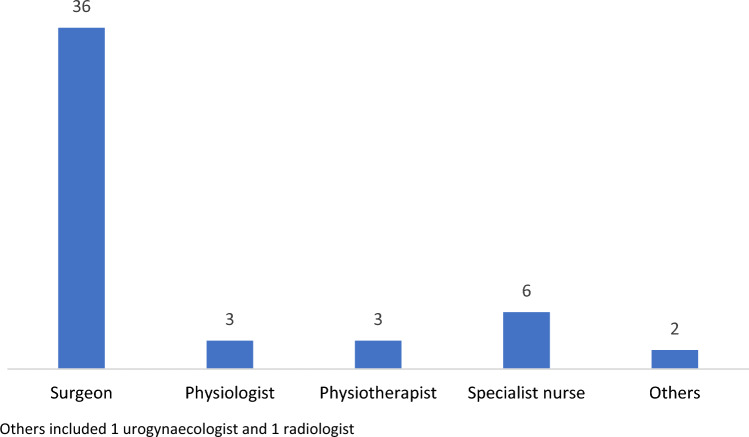
Participants’ clinical role (*n* = 50). Others included 1 urogynaecologist and 1 radiologist

**Fig. 3:**
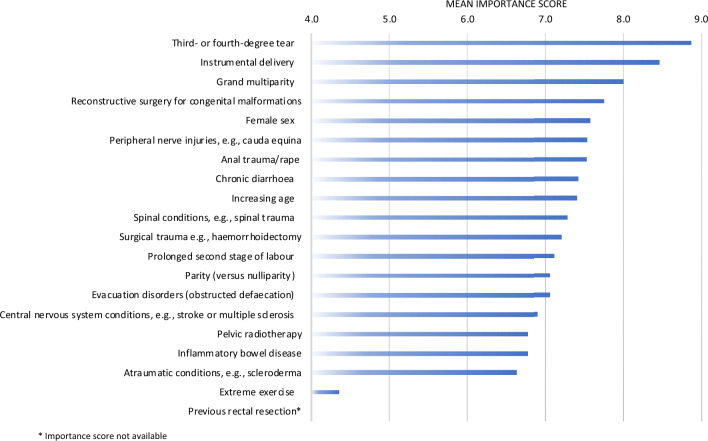
Mean importance score for independent risk factors for faecal incontinence. *Importance score not available

In reviewing recent Delphi studies conducted in the field of coloproctology (Supplement A, Fig. 1), most conducted between two and three rounds of voting (Supplement A, Fig. 2), with modification of survey items between rounds (Supplement A, Fig. 3). This current study included two rounds of anonymous voting by online surveys, followed by a final round of anonymous voting within a consensus meeting (mixed face-to-face and virtual) as illustrated in Fig. 1.

The first-round questionnaire (Supplement B) was developed by PC, MH, and CK on the basis of a pilot survey carried out at TPFS annual meeting in November 2019 and a review of current literature. Potential risk factors were long-listed from textbooks [11–14], the most recent systematic reviews on the prevalence of constipation [15] and faecal incontinence [1, 6] (including an evaluation of the original studies included in these reviews), and a literature search for new studies published since the systematic reviews up to the 1 May 2021. The final selection of potential risk factors for inclusion in this study was determined by the senior author to produce a feasible questionnaire. The questionnaire was divided into three sections: participants’ characteristics, potential risk factors for faecal incontinence, and potential risk factors for chronic constipation. The first section captured the participant’s clinical role, years of experience in that role, and the volume of patients seen with disorders of defaecation in an average month.

For potential risk factors, each item could be rated by the participant as an independent risk factor, a co-factor, or ‘not a risk factor’. An independent risk factor was defined as a risk factor which can increase the risk of faecal incontinence or chronic constipation in an individual even if there are no other risk factors present. For a co-factor to increase the risk, an individual must also have at least one other risk factor. Classification to ‘not a risk factor’ was based on the belief that it neither increased the risk of faecal incontinence or chronic constipation alone or in combination with other factors. All risk factors classified as independent were then rated by importance using a score between 1 (not important) to 10 (critically important) on a Likert scale.

The second and third rounds were dependent on the results of the first and second rounds, respectively. Any risk factor which reached consensus was not carried forward into the subsequent round; any rating option with 10% of the votes or fewer were removed in the subsequent round; new items from participants’ suggestions were reviewed and added to the subsequent round as appropriate (see Supplementary A, Table 1). At the third-round consensus meeting, the results from the previous rounds were presented along with a summary of evidence. Participants voted on the basis of real-time presentation of the results. If consensus was not reached, the participants were invited to have a discussion followed by a re-vote, which was then accepted as final.

#### Anonymous process

Anonymous online tools were used to conduct the surveys (www.onlinesurveys.ac.uk) and the live voting (www.mentimeter.com). The study was co-ordinated by non-voting members of the research team for impartiality.

### Definition of consensus

**Fig. 4:**
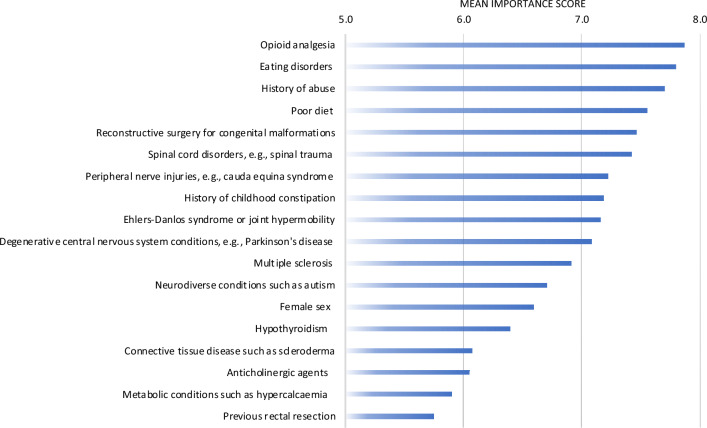
Mean importance score for independent risk factors for chronic constipation

This study used an agreement level of 70% or greater to define consensus. Percentage agreement is a recognised approach to define consensus [8] and 70% agreement or greater is a commonly used parameter in recent Delphi studies within the field of coloproctology (see Supplement A, Fig. 4).

### Statistical analysis

Classification outcomes were presented as counts and percentages. Importance ratings were presented as mean and standard deviation. Statistical analysis was performed using Microsoft Excel, version 16.58 (Microsoft Corporation, Redmond, WA, USA).

### Ethical approval

The study was approved by the Queen Mary Ethics of Research Committee (QMERC20.228).

## Results

### Participant characteristics

Fifty members of TPFS participated in the first round of the study (response rate of 14.7%). Participant clinical roles are summarised in Fig. 2 with the majority being colorectal surgeons (*n* = 36; 72%). Median experience was 15 (8–20) years and the number of patients with faecal incontinence and chronic constipation seen in an average month by participants was 10 (5–20) and 15 (10–20), respectively. Thirty-one members participated in the second round of the study and nine members participated in all three rounds.

### Classification of risk factors

#### Faecal incontinence

In the first round, 28 potential risk factors for faecal incontinence based on long-listing were evaluated (Table 1; Supplement A, Table 2). Seven were considered independent risk factors, including third- or fourth-degree tear (96% agreement), instrumental delivery (92% agreement), parity (70% agreement), grand multiparity (72% agreement), congenital conditions such as Hirschsprung disease or anorectal agenesis (80% agreement), peripheral nerve injury such as cauda equina syndrome (84% agreement), and spinal conditions such as spinal trauma (80% agreement). Two additional items (anal trauma, medications which may cause diarrhoea) were added by participant suggestion.

**Table 1:** Evaluation of potential risk factors for faecal incontinence

Potential risk factor	Agreement (%)		
	Not a risk factor	Co-factor	Independent
Socio-demographic and lifestyle			
Increasing age	–	16	84
Intensive exercise	22	–	78
Female sex	–	29	71
Institutional living	–	90	10
Dietary factors	–	87	13
Obesity	0	86	14
Excessive alcohol consumption	10	74	16
Ethnicity	97	3	–
Unemployment	90	10	–
Low socioeconomic status	71	29	0
Obstetric			
Third- or fourth-degree tear	0	4	96
Instrumental delivery	2	6	92
Prolonged second stage of labour		13	87
Grand multiparity	2	26	72
Parity (versus nulliparity)	4	26	70
High birth weight babies	–	100	0
Episiotomy, first- or second-degree tear	100	0	0
Sphincteric			
Surgical trauma such as haemorrhoidectomy, internal sphincterotomy	–	7	94
Anal trauma/rape (not consensual anal intercourse)*	0	13	87
Atraumatic conditions such as scleroderma or idiopathic internal sphincter atrophy	–	19	81
Reconstructive surgery for congenital malformations such as Hirschsprung or anorectal agenesis	0	20	80
Extra-sphincteric			
Pelvic radiotherapy	–	0	100
Inflammatory bowel disease	0	0	100
Previous rectal resection	0	0	100
Evacuation disorders (obstructed defaecation)	–	11	89
Peripheral nerve injuries such as cauda equina syndrome	0	16	84
Chronic diarrhoea	–	19	81
Spinal conditions such as spinal trauma	0	20	80
Central nervous system conditions such as stroke or multiple sclerosis	–	26	74
Medications which may cause diarrhoea*	0	100	0
Neurodiverse conditions such as autism**	0	100	0
Diabetes mellitus	–	74	26
Depressive disorders	100	0	0

*Added following round 1

**Added following round 3

In the second round, 23 potential risk factors for faecal incontinence were evaluated. Eight were categorised as independent risk factors including increasing age (84% agreement), female sex (71% agreement), prolonged second stage of labour (87% agreement), surgical trauma such as haemorrhoidectomy or lateral sphincterotomy (94% agreement), anal trauma or rape (87% agreement), atraumatic conditions such as scleroderma or idiopathic internal sphincter atrophy (81% agreement), chronic diarrhoea (81% agreement), and central nervous system conditions such as stroke or multiple sclerosis (74% agreement). Four were classified as co-factors, and three were considered ‘not risk factors’ for faecal incontinence, including ethnicity (97% agreement), unemployment (90% agreement), and low socioeconomic status (71% agreement).

In the third round, 11 potential risk factors were evaluated (including 3 new items generated in the consensus meeting). Five were considered independent risk factors including extreme exercise (78% agreement), pelvic radiotherapy (100%), inflammatory bowel disease (100% agreement), previous rectal resection (100% agreement), and evacuation disorders (89% agreement). Four were considered co-factors, and episiotomy, first- or second-degree tear (100% agreement), and depressive disorders (100% agreement) were considered not risk factors for faecal incontinence.

#### Chronic constipation

In the first round, 35 long-listed risk factors for chronic constipation were evaluated (Table 2; Supplement A, Table 3). Five were classified as independent risk factors, including degenerative central nervous conditions such as Parkinson’s disease (76% agreement), peripheral nerve injuries such as cauda equina syndrome (76% agreement), spinal cord disorders such as spinal trauma (74% agreement), previous reconstructive surgery for Hirschsprung disease or congenital anorectal malformation (74% agreement), and opioid analgesia (88% agreement). Two additional items (history of childhood constipation, family history of constipation) were added from participants’ suggestions.

**Table 2:** Evaluation of potential risk factors for chronic constipation

Potential risk factor	Agreement (%)		
	Not a risk factor	Co-factor	Independent
Socio-demographic and lifestyle			
Poor diet	–	10	90
Female sex	0	13	88
Increasing age	–	100	0
Shift work	10	87	3
Obesity	–	84	16
Lack of exercise	–	81	19
Low socioeconomic status	19	81	0
Institutional living	–	78	22
Ethnicity	87	13	–
Unemployment	71	29	–
Medical history			
Metabolic conditions such as hypercalcaemia	–	0	100
Connective tissue diseases such as scleroderma	–	11	89
Ehlers-Danlos syndrome or joint hypermobility syndrome	–	16	84
History of childhood constipation*	0	23	77
Hypothyroidism	–	29	71
Diabetes mellitus	16	84	–
Pregnancy	–	71	29
Family history of constipation*	100	–	–
Neurological conditions			
Multiple sclerosis	–	7	94
Degenerative central nervous system conditions such as Parkinson’s disease	2	22	76
Peripheral nerve injuries such as cauda equina syndrome	0	24	76
Spinal cord disorders such as spinal trauma	0	26	74
Cognitive impairment (any cause)	–	78	22
Previous stroke	–	77	23
Mental health			
Eating disorders	–	7	94
History of abuse (sexual, physical, or neglect)	–	10	90
Neurodiverse conditions such as autism**	–	22	78
Severe endogenous depression	–	87	13
Surgical history			
Previous rectal resection	0	0	100
Reconstructive surgery for congenital malformations such as Hirschsprung or anorectal agenesis	4	22	74
Previous gynaecological surgery	–	78	22
Previous abdominal surgery such as appendicectomy or cholecystectomy	89	11	–
Medications			
Anticholinergic agents	–	0	100
Opioid analgesia	0	12	88
Calcium channel blockers	16	81	3
5-HT_3_ receptor antagonist	19	74	7
Bile acid sequestrants	23	71	7
Cation-containing agents	13	71	16

*Added following round 1

**Added following round 3

In the second round, 32 potential risk factors for chronic constipation were evaluated. Seven were categorised as independent risk factors, including poor diet (90% agreement), Ehlers-Danlos syndrome or joint hypermobility syndrome (84% agreement), hypothyroidism (71% agreement), history of childhood constipation (77% agreement), multiple sclerosis (94% agreement), eating disorders (94% agreement), and history of abuse (90% agreement). Twelve were considered co-factors, and ethnicity (87% agreement) was considered not a risk factor for chronic constipation.

In the third round, 13 potential risk factors remained without consensus (including 1 new item generated in the consensus meeting). Six were considered independent risk factors, including female sex (88% agreement), metabolic conditions such as hypercalcaemia (100% agreement), connective tissue diseases such as scleroderma (89% agreement), neurodiverse conditions such as autism (78% agreement), previous rectal resection (100% agreement), and anticholinergic agents (100% agreement). Four were considered co-factors and three were considered not risk factors for chronic constipation, including unemployment (71% agreement), family history of constipation (100% agreement), and previous abdominal surgery such as appendicectomy or cholecystectomy (89% agreement).

### Importance rating of risk factors

#### Faecal incontinence

The independent risk factors considered most important for faecal incontinence were third- and fourth-degree tear (mean importance score of 8.88 ± 1.57), instrumental delivery (mean importance score of 8.47 ± 15.8), and grand multiparity (mean importance score of 8.00 ± 1.63) (Fig. 3). The independent risk factor considered least important was extreme exercise (mean importance score of 4.35 ± 2.26). Detailed results including the mean importance scores (± SD) are provided in Supplement A, Table 4.

#### Chronic constipation

The independent risk factors considered most important for chronic constipation were opioid analgesia (mean importance score of 7.87 ± 2.05), eating disorders (mean importance score of 7.80 ± 1.72), and history of abuse (mean importance score of 7.70 ± 1.89) (Fig. 4). The independent risk factor considered least important was previous rectal resection (mean importance score of 5.75 ± 2.47). Detailed results including the mean importance scores (± SD) are provided in Supplement A, Table 5.

## Discussion

To our knowledge, this is the first study to comprehensively evaluate the importance of potential risk factors for benign disorders of defaecation using Delphi methodology. Consensus was achieved for classification (independent risk factor, co-factor, not a risk factor) of all potential risk factors evaluated (33 for faecal incontinence and 38 for chronic constipation). Mean importance scores were also produced for 19 of the 20 independent risk factors for faecal incontinence and all the 18 independent risk factors for chronic constipation.

Age and sex were the most evaluated risk factors for faecal incontinence and chronic constipation in the literature. While female sex was considered an independent risk factor for faecal incontinence and chronic constipation, increasing age was classified as an independent risk factor for faecal incontinence but a co-factor for chronic constipation. Meta-analyses of studies reporting the prevalence of chronic constipation in the general population have reported a significant association with female sex [4, 16, 17]. Barberio et al. [2] reported higher pooled prevalence of functional constipation in women compared with men, irrespective of the Rome definition used. The evidence for increasing age as a risk factor for chronic constipation is less consistent. While Suares and Ford [15] reported a modest increase in the pooled prevalence (17% in the ≥ 60 years compared with 12% in the < 29 years) and risk of chronic constipation in the higher age group (OR of 1.41 in the ≥ 60 years compared with < 29 years as baseline), Barberio et al. [2] found no statistical differences in the prevalence of functional constipation between the different age groups. A recent population survey even showed the highest prevalence of Rome IV functional constipation in the youngest age group (9.9% in those aged 18–29 years) [17]. Evidence from population studies largely supports the association between faecal incontinence and age and female sex [6, 18, 19].

The independent risk factors considered most important through Delphi approach for faecal incontinence were obstetric factors, including third- or fourth-degree tears (i.e. obstetric anal sphincter injury), instrumental delivery, and grand multiparity. Several systematic reviews have concluded that obstetric anal sphincter injury is significantly associated with an increased risk of anal [20–22] and faecal incontinence [22]. A meta-analysis by Cattani et al. [22] demonstrated a significant risk of anal incontinence associated with forceps delivery (OR 1.35 [CI 1.12–1.63]) and vacuum extraction delivery (OR 1.17 [CI 1.04–1.31]). The evidence of association between multiparity and anal or faecal incontinence is equivocal, with several studies reporting a significant association [23–25] and others suggesting the contrary [19, 26].

The current study did not consider episiotomy and first- or second-degree tears to be a risk factor for faecal incontinence. Several systematic reviews and meta-analyses have been performed to assess the risk of anal or faecal incontinence associated with episiotomy. LaCross et al. [20] suggested an increased risk of anal incontinence (OR 1.74 [CI 1.28–2.38]) in women who had an episiotomy; however, Bols et al. [21] did not find any significant association between first- or second-degree tear with faecal incontinence. Cattani et al. [22] described an increased risk of anal (OR 1.51 [CI 1.16–1.96], *p* = 0.002) but not faecal (OR 1.11 [CI 0.36–3.41], *P* = 0.85) incontinence when an episiotomy is performed. Interpretation of these findings is challenging because of heterogeneity in episiotomy practice (routine vs. selective) and type of episiotomy performed (median vs. mediolateral).

One of the most important independent risk factors for chronic constipation was history of abuse, but no large population study has examined this risk factor, most likely because of its sensitive nature. Several small observational studies have consistently reported a significant association between history of abuse and constipation [27], functional evacuation disorder [28, 29], symptoms of incomplete evacuation [30], or multiple pelvic floor complaints [31].

Potentially modifiable risk factors for faecal incontinence and chronic constipation included dietary factors and obesity. Dietary factors were considered a co-factor for faecal incontinence and an independent risk factor for chronic constipation. A systematic review by Colavita and Andy [32] found very limited data on the role of diet in the pathogenesis of faecal incontinence. Only one out of four studies found an association between low dietary fibre and faecal incontinence, but all five studies which assessed the effectiveness of diet as a treatment for faecal incontinence showed that fibre supplement improved faecal incontinence symptoms. Studies that have evaluated the dietary differences between individuals with and without chronic constipation have found a significant association with fluid intake [16, 33, 34], but evidence of association between low dietary fibre and chronic constipation is equivocal [35]; however, several systematic reviews have surmised that fibre supplementation is an effective treatment for chronic constipation [36, 37]. Obesity was considered a co-factor for both faecal incontinence and chronic constipation. Evidence from several observational studies have found a significant association between obesity and faecal [19, 26, 38–40] or flatus incontinence [38, 41], which may be due to an increased risk of loose stools [39] or the use of medications for weight loss [42] or diabetes [43]. Though some studies have reported no significant relationship [44] or an inverse relationship [16] between obesity and chronic constipation when defined as hard or infrequent stools, several studies have suggested an association between obesity and difficulty in rectal evacuation [39, 41]. Other modifiable risk factors for faecal incontinence included diarrhoea, evacuation disorder, diabetes, medications, and excessive alcohol consumption. Other modifiable risk factors for chronic constipation included eating disorders, diabetes, hypothyroidism, lack of exercise, and medications.

Diarrhoea (or loose stools) was considered one of the most important risk factors for faecal incontinence by several observational studies [18, 19, 26] but our experts assigned less importance to this risk factor. This discrepancy likely reflects the clinical practice of our experts, who were predominantly British colorectal surgeons, and may differ from opinions of patients and other specialists (general practitioner and gastroenterologists). This is a significant limitation on the generalisability of our findings. Further, although our results may be relevant to other developed countries with similar populations and risk factors, they may not be applicable to countries with less economic and healthcare resources. Acknowledging that the Delphi technique has been criticised for the quality of scientific evidence, the validity of the results, and the inconsistency of the study design [9, 10], we generated the questionnaire from scientific evidence published in peer-reviewed journals and were rigorous in pre-defining and adhering to the core elements of the Delphi process. Though the first round of this study was a structured round, which is a deviation from the ‘classical’ Delphi approach [10], this is common practice within clinical Delphi studies [9] and is consistent with recent Delphi publications within the field of coloproctology (Supplement A). The opportunity for ‘experts’ to freely express their opinion was maintained by the provision of free-text suggestions. The method enabled the importance of risk factors with low volume of evidence due to sensitive nature, e.g. history of abuse, or low prevalence within the general population, e.g. anal trauma, to be evaluated in the same manner as well-established risk factors such as age. There was an attrition rate between rounds (41% from round 1 to round 2, and 71% from round 2 to round 3), which is not unexpected in a Delphi study [45]. Despite this, we were able to maintain a multidisciplinary representation of experienced practitioners, which included colorectal surgeons, clinical nurse specialists, gastrointestinal physiologists, and a radiologist, from the first to the final round. Finally, this study did not include patients in the participants as it would be not have been possible to ensure unbiased and up to date clinical knowledge across all participants.

## Conclusion

This study has highlighted some risk factors that may be modifiable in terms of prevention or treatment. The results will be used to inform a Bayesian risk prediction tool to assist clinical assessment of a patient’s risk of developing disorders of defaecation.

## Supplementary Information

Below is the link to the electronic supplementary material.

Supplementary file1 (XLSX 111 KB)

Supplementary file2 (PDF 122 KB)

## Data Availability

The data that support the findings of this study are available from the corresponding author, PC, upon reasonable request.
