# Supplementary material for: Evaluating the risk factors for the development of benign disorders of defaecation: a surgical perspective
Source: Tech Coloproctol. 2023 Jul 27;27(10):847–57. doi: 10.1007/s10151-023-02843-w (PMC10484816; doi:10.1007/s10151-023-02843-w)
Supplement: Supplementary file 2 — Supplementary file2 (PDF 122 KB) [file 10151_2023_2843_MOESM2_ESM.pdf]

Evaluating the influence of risk factors on the development of defaecatory problems: A Delphi study

Page 1: Welcome to the Survey

Problems of defaecation are common and largely divided into chronic constipation and faecal incontinence, with a significant proportion of individuals experiencing both. An understanding of the risk factors underpinning the problems of defaecation is important as it may improve our understanding of the pathophysiology and provide targets for disease prevention. Several risk factors for developing chronic constipation and faecal incontinence have been identified, predominantly through large cross-sectional studies. However, these studies are heterogenous in several aspects: the definitions of chronic constipation and faecal incontinence, their methodology, the study population, and the potential risk factors assessed. This major limitation prevents the aggregation of results by meta-analysis and so the true impact of individual risk factors on the development of chronic constipation and faecal incontinence remains unknown.

The Delphi technique is an effective method in arriving at consensus on broad and complex problems. Using the clinical experience and knowledge of experts in the field of pelvic floor disorders in combination with the existing body of evidence, a Delphi study can derive a consensus on what the most important risk factors are for the development of faecal incontinence and chronic constipation, in a focus and cost-effective manner.

You are being invited to participate in this survey because of your expertise in the field of pelvic floor disorders as members of the Pelvic Floor Society. This survey forms part of a research study titled 'Evaluating the influence of risk factors on the development of defaecatory problems: A Delphi study'. This study is being performed by Miss Pam Chaichanavichkij under the supervision of Professor Charles Knowles from the Bilzard Institute, School of Medicine and Dentistry, at Queen Mary University of London. The main objective of this study is to identify the most important risk factors for the development of faecal incontinence and constipation, and to determine if a risk factor is independent or has to co-exist with other risk factors in order to influence disease development.

This survey forms the first round of a Delphi study, which will consist of 2 rounds of surveys and 1 final round of a consensus meeting. Participants who take part in all 3 rounds will be acknowledged in any publications of this Delphi study, provided that they give consent to be identifiable in the generated

materials. The survey contains 13 questions, consisting of 3 questions on your experience in this field, 4 questions on the risk factors for faecal incontinence and 6 questions on the risk factors for chronic constipation. The survey should take a maximum of 30 minutes to complete.

If you are interested in taking part, please download a copy of the participant information sheet here and retain this for your records before starting the survey. If you have any questions, please contact the researcher at p.chaichanavichkij@qmul.ac.uk.

Your participation is entirely voluntary, and you can withdraw at any time. This survey is anonymous. No identifiable information is collected in this survey. The record of your survey responses does not contain any identifying information about you.

Queen Mary Ethics of Research Committee reference number: QMERC20.228

Researchers:

Miss Pam Chaichanavichkij, Miss Morghan Hartmann,

Dr. Scott McLachlan, Professor Norman Fenton, and Professor Charles Knowles.

If you have chosen to complete a printed version of this survey, please send your response to:

Miss Pam Chaichanavichkij; The Blizard Institute, Abernathy building, 4 Newark Street, London E1 2AT.

Page 2: Survey Responder

This section seeks to understand the survey responder's clinical background and experience. If your current situation has changed as a result of the COVID-19 pandemic, please answer these questions with reference to your clinical practice prior to the COVID-19 pandemic.

1. Please select your clinical role * *Required*

More info

- Surgeon
- Gastroenterologist
- Specialist nurse
- Physiotherpist
- GI physiologist
- Other

1.a. If you selected Other, please specify:

2. Please indicate the number of years of experience in this role * *Required*

More info

3. In an average month, how many patients with the following defaecatory problems do you see?

More info

Constipation	
Faecal incontinence	

Page 3: Risk factors for faecal incontinence

These questions seek to understand the significance of each factor as an independent risk factor for faecal incontinence.

The individual item within each question refers to a potential risk factor for faecal incontinence, which were identified from the pilot questionnaire and the scoping literature review.

Each possible risk factor should be rated as exactly one of the folowing possible types:

- Independent: this risk factor alone increases the risk of faecal incontinence even if there are no other risk factors.
- Co-factor only: for this risk factor to increase the risk of faecal incontinence there must also be at least one other risk factor.
- Not a risk factor: this risk factor does not increase the risk of faecal incontinence.

Independent risk factors require a rating on its importance as a risk factor:

- A score of 0: not a risk factor
- A score of 1: not an importnant risk factor
- A score of 5: a moderately important risk factor
- A score of 10: a critically important risk factor

For any item that is *not a risk factor* or a *co-factor only*, no rating for importance is required.

4. Please rate the type and importance of the following socio-demographic and lifestyle factors as a risk factor for faecal incontinence.

More info

	Type of risk factor ✱ *Required*			Importance									
	Not a risk factor	Cofactor	Independent risk factor	1	2	3	4	5	6	7	8	9	10
Increasing age	○	○	○	○	○	○	○	○	○	○	○	○	○
Female gender	○	○	○	○	○	○	○	○	○	○	○	○	○
Low socioeconomic status	○	○	○	○	○	○	○	○	○	○	○	○	○
Unemployment	○	○	○	○	○	○	○	○	○	○	○	○	○
Institutional living	○	○	○	○	○	○	○	○	○	○	○	○	○
Dietary factors	○	○	○	○	○	○	○	○	○	○	○	○	○
Intensive exercise	○	○	○	○	○	○	○	○	○	○	○	○	○

Obesity	○	○	○	○	○	○	○	○	○	○	○	○	○
Excessive alcohol consumption	○	○	○	○	○	○	○	○	○	○	○	○	○
Ethnicity	○	○	○	○	○	○	○	○	○	○	○	○	○

4.a. Other (please specify)

5. Please rate the type and importance of the following obstetric factors as a risk factor for faecal incontinence in parous women.

More info

	Type of risk factor ✱ *Required*			Importance									
	Not a risk factor	Cofactor	Independent risk factor	1	2	3	4	5	6	7	8	9	10
Parity (versus nulliparity)	○	○	○	○	○	○	○	○	○	○	○	○	○
Grand multiparity	○	○	○	○	○	○	○	○	○	○	○	○	○
Instrumental delivery	○	○	○	○	○	○	○	○	○	○	○	○	○
High birthweight babies	○	○	○	○	○	○	○	○	○	○	○	○	○
Episiotomy, 1st or 2nd degree tear	○	○	○	○	○	○	○	○	○	○	○	○	○
3rd or 4th degree tear	○	○	○	○	○	○	○	○	○	○	○	○	○
Prolonged second stage of labour	○	○	○	○	○	○	○	○	○	○	○	○	○

5.a. Other (please specify)

6. Please rate the type and importance of the following sphincteric factors as a risk factor for faecal incontinence.

	Type of risk factor ✱ *Required*			Importance									
	Not a risk factor	Cofactor	Independent risk factor	1	2	3	4	5	6	7	8	9	10
Congenital conditions such as Hirschsprung's, anorectal agenesis													
Surgical trauma such as haemorrhoidectomy, internal spincterotomy													
Atraumatic conditions such as scleroderma, idiopathic internal sphincter atrophy													

6.a. Other (please specify)

7. Please rate the type and importance of the following extrasphincteric factors as a risk factor for faecal incontinence.

More info

	Type of risk factor ✱ *Required*			Importance									
	Not a risk factor	Cofactor	Independent risk factor	1	2	3	4	5	6	7	8	9	10

Evacuation disorders (obstructed defaecation)													
Diabetes mellitus													
Conditions causing rectal inflammation													
CNS conditions such as stroke, MS													
Spinal conditions such as spinal trauma													
Peripheral nerve injuries such as cauda equina syndrome													
Psychiatric or behavioural disorders													
Chronic diarrhoea													

7.a. Other (please specify)

Page 4: Risk factors for chronic constipation

These questions seek to understand the significance of each factor as an **independent risk factor for chronic constipation**.

The individual item within each question refers to a potential risk factor for chronic constipation, which were identified from the pilot questionnaire and the scoping literature review.

Each possible risk factor should be rated as exactly one of the folowing possible types:

- Independent: this risk factor alone increases the risk of chronic constipation even if there are no other risk factors.
- Co-factor only: for this risk factor to increase the risk of chronic constipation there must also be at least one other risk factor.
- Not a risk factor: this risk factor does not increase the risk of chronic constipation.

Independent risk factors require a rating on its importance as a risk factor:

- A score of 1: not an importnant risk factor
- A score of 5: a moderately important risk factor
- A score of 10: a critically important risk factor

For any item that is *not a risk factor* or a *co-factor only*, no rating for importance is required

8. Please rate the type and importance of the following socio-demographic and lifestyle factors as a risk factor for chronic constipation.

More info

	Type of risk factor ✱ *Required*			Importance									
	Not a risk factor	Cofactor	Independent risk factor	1	2	3	4	5	6	7	8	9	10
Increasing age	○	○	○	○	○	○	○	○	○	○	○	○	○
Female gender	○	○	○	○	○	○	○	○	○	○	○	○	○
Low socioeconomic status	○	○	○	○	○	○	○	○	○	○	○	○	○
Unemployment	○	○	○	○	○	○	○	○	○	○	○	○	○
Shift-work	○	○	○	○	○	○	○	○	○	○	○	○	○
Institutional living	○	○	○	○	○	○	○	○	○	○	○	○	○
Poor diet	○	○	○	○	○	○	○	○	○	○	○	○	○

Lack of exercise													
Obesity													
Ethnicity													

8.a. Other (please specify)

9. Please rate the type and importance of the following medical conditions as a risk factor for chronic constipation.

More info

	Type of risk factor ✱ *Required*			Importance									
	Not a risk factor	Cofactor	Independent risk factor	1	2	3	4	5	6	7	8	9	10
Diabetes mellitus													
Hypothyroidism													
Pregnancy													
Metabolic conditions such as hypercalcaemia													
Connective tissue diseases such as scleroderma													
Ehler-Danlos syndrome or joint hypermobility syndrome													

9.a. Other (please specify)

10. Please rate the type and importance of the following neurological conditions as a risk factor for chronic constipation.

More info

	Type of risk factor ✱ *Required*			Importance									
	Not a risk factor	Cofactor	Independent risk factor	1	2	3	4	5	6	7	8	9	10
Degenerative CNS conditions such as Parkinson's disease	○	○	○	○	○	○	○	○	○	○	○	○	○
Multiple sclerosis	○	○	○	○	○	○	○	○	○	○	○	○	○
Previous stroke	○	○	○	○	○	○	○	○	○	○	○	○	○
Spinal cord disorders such as spinal trauma	○	○	○	○	○	○	○	○	○	○	○	○	○
Peripheral nerve injuries such as cauda equina syndrome	○	○	○	○	○	○	○	○	○	○	○	○	○
Cognitive impairment (any cause)	○	○	○	○	○	○	○	○	○	○	○	○	○

10.a. Other (please specify)

11. Please rate the type and importance of the following mental health conditions as a risk factor for

chronic constipation.

	Type of risk factor ✱ Required			Importance									
	Not a risk factor	Cofactor	Independent risk factor	1	2	3	4	5	6	7	8	9	10
Severe endogenous depression	○	○	○	○	○	○	○	○	○	○	○	○	○
Eating disorders	○	○	○	○	○	○	○	○	○	○	○	○	○
History of abuse (sexual, physical, or neglect)	○	○	○	○	○	○	○	○	○	○	○	○	○

11.a. Other (please specify)

____

12. Please rate the type and importance of the following surgical history as a risk factor for chronic constipation.

	Type of risk factor ✱ Required			Importance									
	Not a risk factor	Cofactor	Independent risk factor	1	2	3	4	5	6	7	8	9	10
Previous abdominal surgery such as appendicectomy, cholecystectomy	○	○	○	○	○	○	○	○	○	○	○	○	○
Previous pelvic surgery such as hysterectomy	○	○	○	○	○	○	○	○	○	○	○	○	○
Previous low anterior resection	○	○	○	○	○	○	○	○	○	○	○	○	○

Previous reconstructive surgery for Hirschsprung's or congenital anorectal malformation	○	○	○	○	○	○	○	○	○	○	○	○	○

12.a. Other (please specify)

13. Please rate the type and importance of the following medications as a risk factor for chronic constipation.

More info

	Type of risk factor ✱ *Required*			Importance									
	Not a risk factor	Cofactor	Independent risk factor	1	2	3	4	5	6	7	8	9	10
5-HT3 receptor antagonist	○	○	○	○	○	○	○	○	○	○	○	○	○
Opiate analgesics	○	○	○	○	○	○	○	○	○	○	○	○	○
Anticholinergic agents	○	○	○	○	○	○	○	○	○	○	○	○	○
Calcium channel blockers	○	○	○	○	○	○	○	○	○	○	○	○	○
Bile acid sequestrants	○	○	○	○	○	○	○	○	○	○	○	○	○
Cation-containing agents	○	○	○	○	○	○	○	○	○	○	○	○	○

13.a. Other (please specify)

By clicking the 'Finish' button below, you are consenting to participate in this study, as it is described in the participant information sheet, which you can download here.

Page 5: End of Survey

Thank you for your participation in this study.

If you have any concerns about the manner in which the study was conducted, in the first instance, please contact the researcher responsible for the study (Professor Charles Knowles: c.h.knowles@qmul.ac.uk). If you have a complaint which you feel you cannot discuss with the researchers then you should contact the Research Ethics Facilitators by e-mail: research-ethics@qmul.ac.uk. When contacting the Research Ethics Facilitators, please provide details of the study title, description of the study and QMERC reference number (where possible), the researcher(s) involved, and details of the complaint you wish to make.
